# Limits of Prediction for Machine Learning in Drug Discovery

**DOI:** 10.3389/fphar.2022.832120

**Published:** 2022-03-10

**Authors:** Modest von Korff, Thomas Sander

**Affiliations:** Idorsia Pharmaceuticals Ltd., Allschwil, Switzerland

**Keywords:** machine learning, drug discovery, extrapolation, data set, PLS (partial least square), Gaussian regression, random forest, support vector regression

## Abstract

In drug discovery, molecules are optimized towards desired properties. In this context, machine learning is used for extrapolation in drug discovery projects. The limits of extrapolation for regression models are known. However, a systematic analysis of the effectiveness of extrapolation in drug discovery has not yet been performed. In response, this study examined the capabilities of six machine learning algorithms to extrapolate from 243 datasets. The response values calculated from the molecules in the datasets were molecular weight, cLogP, and the number of sp3-atoms. Three experimental set ups were chosen for response values. Shuffled data were used for interpolation, whereas data for extrapolation were sorted from high to low values, and the reverse. Extrapolation with sorted data resulted in much larger prediction errors than extrapolation with shuffled data. Additionally, this study demonstrated that linear machine learning methods are preferable for extrapolation.

## Introduction

In drug discovery, new molecules undergo clinical trials in human subjects only after numerous checks for safety and potency in biological test systems. Often, a drug suitable for oral administration is desired, i.e., a molecule that can cross cellular membranes separating the gastrointestinal system and blood vessels. After absorption, blood vessels distribute the molecule throughout the organism and to its site of action. Blood contains many proteins that bind a substantial fraction of any compound. During distribution, molecules pass through the liver, which contains enzymes able to metabolize many types of chemical substances, thus reducing the concentration of the active drug (clearance). An important measure used in the optimization of a bioactive molecule is plasma exposure after oral administration, often expressed as “area under the curve” (AUC), i.e., the concentration of the active molecule in blood plasma integrated over time. Bioavailability depends on multiple properties of the molecule including cell layer permeability and clearance in the liver. When a molecule reaches the target protein, it must bind in such a way that it has the desired effect. A specific assay is usually developed to measure the effect of the molecule on the target protein. At present, it is still not possible to design a successful drug, fulfilling all necessary requirements, without biological tests. However, biological testing requires time and resources, which limit the number of compounds that can be explored. Medicinal chemists require quantitative models allowing prioritization the most promising molecules for biological testing.

### Related Work

The use of quantitative structure-activity relationships (QSAR) is essential in drug discovery and has been investigated in multiple publications (Gramatica, 2007; Cherkasov et al., 2014). Recently, huge efforts has been undertaken to find appropriate meta-parameter for QSAR models (Olier et al., 2018). It is well known that statistical models lose their predictive power when they are outside the range of calibration. Outside the calibration range, confidence intervals become infinite. These limits have been previously discussed for QSAR from (Tong et al., 2005) and were formulated in OECD policies for the validation of QSAR models (Member countries, 2004; OECD, 2014). Closely related to the calibration range is the term applicability domain. The term applicability domain is used in cheminformatics for quantitative structure activity models. The OECD guideline demands to consider the applicability domain but does not give a binding definition. By Roy et al. the application domain was defined as “The AD is a theoretical region in chemical space encompassing both the model descriptors and modeled response which allows one to estimate the uncertainty in the prediction of a particular compound based on how similar it is to the training compounds employed in the model development” (Roy et al., 2015). If the predicted molecules are similar to the training molecules in descriptor space, they are in the application domain (Jaworska et al., 2005). In drug discovery, the modeled response are molecular properties which the medicinal chemists aim to optimise. So, the properties medicinal chemists would like to predict are often outside the range of response values, which were already covered by experiments. At the start of a drug discovery project, a few molecules are usually identified which show modest activity at the target protein site. Starting compounds are modified by medicinal chemists to improve their properties. By adding all available information into the new compounds, they improve their characteristics over time. The next compound is often designed with the aim to show a lower binding constant to the target protein. Usually, this compound is similar to the already synthesized compounds and therefore in the applicability domain. During this optimization process, the desired response values are outside the range of the available response values. A model that aims to support the medicinal chemist in his work needs the capability of extrapolation. Recently, the use of extrapolation through machine learning, to assess the bioactivity of a molecule in drug discovery, has been evaluated (Cortés-Ciriano et al., 2018). Extrapolation outside the upper limits of the measured value range is wanted for the plasma exposure after oral administration. The plasma exposure should be as high as possible, but in a drug discovery project it is often to low. Additionally, frequently the majority of available response values are far away from the desired value range.

### Our Work

The missing information in QSAR literature about differences between the errors of interpolation and extrapolation triggered a question. How effective can extrapolation of response values for chemical molecules be? To answer this question, we decided to use organic molecule datasets with calculated physicochemical properties. The physicochemical properties were used as response values in this study and were calculated from the molecular structure. Mathematically, a molecule is represented as a small graph with colored edges and colored nodes. This molecular graph cannot directly be used as input for the applied machine learning methods. The graph must be transformed into a vector, a chemical descriptor. Machine learning creates models that relate descriptor vectors to the corresponding response values. With our setup a fully correct machine learning model was theoretically possible. The complete information needed to predict the response values was enclosed in the molecular structure. If this information is transferred to the descriptor vector and the machine learning algorithm constructs a perfect fitting model, a correct prediction will result. This model is “semi-mechanistic”, which is covered by the OECD guideline “When the AD is defined in more mechanistic terms, the (Q)SAR can predict reliably beyond the physicochemical and response space of the training set”. In our experimental setup the used response values allowed the machine learning algorithms to create such “semi-mechanistic” models.

## Methods

### Datasets

**FIGURE 1:**
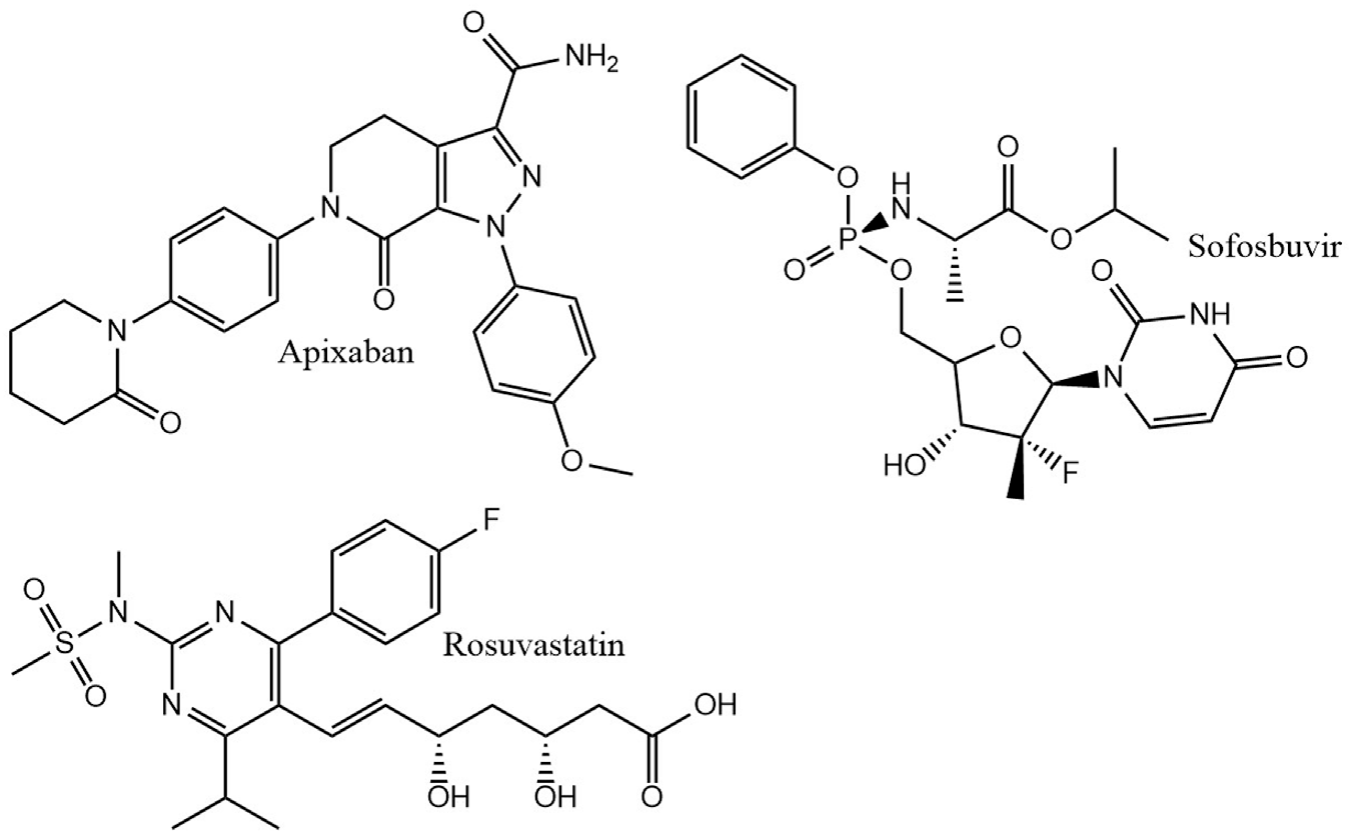
Seed molecules for dataset generation.

For the construction of our molecule datasets, the size and structure of typical datasets in drug discovery was considered. In a drug discovery project, the molecules usually show a high similarity. New molecules are derived from a starting molecule that is explored by medicinal chemists. The newly synthesized molecules are similar to the starting molecule, but ideally have the desired features. We mimicked this process by taking a known drug molecule and removing randomly peripheral non hydrogen atoms. The removed atom was replaced with an appropriate number of hydrogen atoms. Rings were also randomly cut. Three top selling drug molecules were chosen: apixaban, rosuvastatin, and sofosbuvir (Figure 1). From each molecule, three sets, ***S***_***apix,1–3***_, ***S***_***rosu,1–3***_ and ***S***_***sofo,1–3***_, of about 300 molecules each were created. Consequently, nine datasets were constructed from three blockbuster drugs. Similar molecules are needed for successful machine learning models in QSAR (Netzeva et al., 2005). The similarity of test- and training molecules was guaranteed by our molecule degradation approach.

### Dependent Variables

**FIGURE 2:**
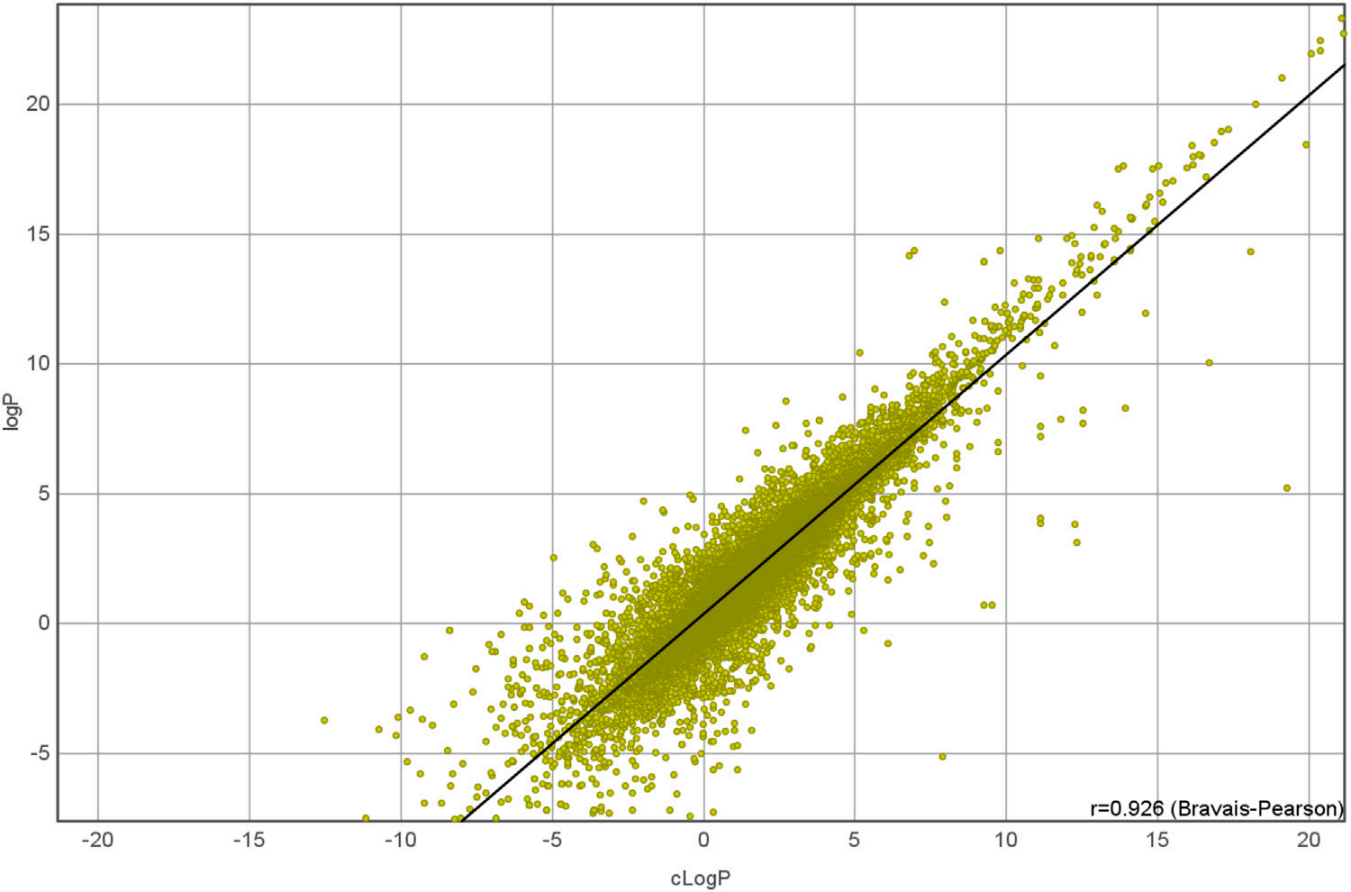
Comparison of 25,000 experimental logP values with DataWarrior calculated logP.

Dependent variables and response variables were calculated for each molecular structure. The simplest dependent variable in this study was molecular weight, which was calculated from the corresponding molecular formula. The logP value, the logarithm of the 1-octanol/water partition coefficient, is a more sophisticated variable which estimates the distribution of a drug based on an octanol/water system. The cLogP value assesses the permeation of a molecule from the gastrointestinal tract into blood vessels, and it is an important measure in drug discovery. Here, a fragmental approach from DataWarrior (Sander et al., 2015) was used to calculate the cLogP. This fragmental approach was developed for the OSIRIS Property Explorer (OsirisP) and successfully benchmarked in a large study with 90,000 compounds (Mannhold et al., 2009). In this independent examination, OsirisP ranked between the top logP calculation methods. An improved version of the Osiris logP calculator was implemented in DataWarrior in 2014. This updated OsirisP calculation is implemented as increment system adding contributions of every atom based on its atom type. OsirisP distinguishes around 400 atom types. This includes hybridisation state, ring membership, aromaticity, and additionally to the older version charges. More than 5,000 compounds with experimentally determined logP values were used as training set to calculate the increments. A recent comparison with 25,000 experimental logP values is given in Figure 2. The strong relation between the experimental and the calculated logP is shown by a correlation coefficient of 0.93. However, this strong correlation is not needed for our experimental setup. Important for the experiment is the linear dependency between the molecular structure and the calculated logP values. Theoretically, this linear dependency allows linear regression methods like partial least square regression a perfect fit of dependent and independent variables.

A third response variable was the number of sp3-carbon atoms in a molecule, where each sp3-carbon atom has 4 neighboring atoms. In early drug discovery, the number of sp3-carbon atoms is used to chose molecules for high throughput screening in biological assays. For every molecule in the nine datasets ***S***_***apix,1–3***_, ***S***_***rosu,1–3***_ and ***S***_***sofo,1–3***_, the three dependent variables were calculated. By considering independent and dependent variables, a set of 27 datasets was obtained. A summary of the obtained values is given in Table 1.

**TABLE 1:** Summary of the response values for all datasets. The first column indicates the property and the other columns the minimum, maximum, average, standard deviation, and median values.

	min	max	avr	sdv	median
Apixaban					
MW	110	434	288	101	294
sp3-atoms	2	19	10	3	10
cLogP	−2.5	6.8	2.1	1.6	2.2
Rosuvastatin					
MW	60	453	255	115	256
sp3-atoms	0	17	8	4	8
cLogP	−1.0	6.0	2.6	1.3	2.6
Sofosbuvir					
MW	110	500	317	108	317
sp3-atoms	5	24	16	4	16
cLogP	−4.8	4.8	0.8	1.2	0.9

### Descriptors

A molecular graph is inappropriate input for most machine learning algorithms. Molecular descriptors are used in cheminformatics to describe molecular structure in algebraic form (Todeschini and Consonni, 2008). For a descriptor, a molecular graph is usually converted into a vector, which is the input for machine learning. The transformation from a molecule into a vector is one directional and comes with a loss of information. The molecular structure can not be recovered from the vector. Different transformations result in different losses of information. For this reason, three different topological molecule descriptors were chosen.

### Fragment Fingerprint Descriptor

The fragment fingerprint is a dictionary based descriptor with a length of 512 bits. Each bit represents a substructure fragment. The dictionary of 512 substructures was created by a computational procedure, which had been optimized to achieve two goals: 1) any of these fragments should occur frequently in organic molecule structures and 2) each fragment should be linearly independent with regard to their substructure-match-pattern in diverse organic compound sets. To generate a descriptor vector, the molecular structure is searched for any of the substructures in the dictionary. For any match, the corresponding bit of the vector is set to 1. Any molecular structure is represented by a binary vector of length 512. The fragment fingerprint descriptor belongs to the same class as the “MDL structure keys” (McGregor and Pallai, 1997), which have recently been shown to outperform 3D descriptors in virtual screening (Nettles et al., 2006).

### Path Fingerprint Descriptor

The path fingerprint is a molecular graph path walking fingerprint descriptor. All distinguishable paths with up to 7 atoms are hashed into a descriptor vector of 512 bits. This descriptor is conceptually similar to ChemAxCFp, the chemical fingerprints from ChemAxon (ChemAxon, 1998) and to the Daylight fingerprints (Daylight, 1998).

### Skeleton Spheres Descriptor

The skeleton spheres descriptor is a vector of integers which counts the occurrence of different substructures in a molecule. Five circular layers with increasing bond distance are located for each atom in the molecule. Hydrogen atoms are not considered. This results in four fragments starting with the naked central atom, adding one layer at a time. Every fragment is encoded as a canonical string (id-code), similar to the generation of canonical SMILES (Weininger et al., 1989). The canonical id-code includes the stereochemistry of the encoded fragment, which is a feature missing in other molecular descriptors. The id-code is then assigned to one of 1,024 fields in a vector. Therefore, the hash value of the id-code is calculated and the corresponding value in the vector is increased by one. The Hashlittle algorithm (Jenkins, 2006) is used as a binning function, which takes a text string as input and returns an integer value between 0 (inclusive) and 1,024 (exclusive). In preliminary experiments, this hash function showed a good uniform distribution of the generated hash values. To consider the molecular scaffold without the influence of the heteroatoms, the whole calculation is repeated while replacing the hetero atoms with carbon. The resulting hash values are used to increment the corresponding fields in the vector. By adding this skeleton information to the descriptor vector, the similarity calculation between two descriptor vectors becomes a bit insensitive to the exact position of the heteroatoms in two molecules. This directs the similarity value towards the perception of similarity by medicinal chemists. For medicinal chemists, the exact position of a hetero atom is not as discriminating as it would be for the spheres descriptor without the skeleton coding part. The additional consideration of the scaffold information and the use of a histogram instead of a binary vector distinguishes the skeleton spheres descriptor from other circular fingerprints. (Glem et al., 2006).

Each of the nine molecule sets ***S***_***apix,1–3***_, ***S***_***rosu,1–3***_, and ***S***_***sofo,1–3***_ was compiled into three descriptor sets fragment fingerprint, path fingerprint and skeleton spheres.

### Dataset Construction

A dataset ***D*** contains a matrix ***X*** and a vector ***y***. Every row in the matrix ***X*** represents a molecule by one of the three descriptors, fragment fingerprint, path fingerprint, and skeleton spheres. Corresponding to a row *i* in ***X*** is a response value *i* in ***y***. Three response values, molecular weight, cLogP and sp3-carbon, were available for each row in ***X***. In drug discovery projects, the optimization process aims for response values outside the range of response values initially obtained. To assess the predictive power of a machine learning tool in a drug discovery project, we sorted the compounds by their response values. One dataset contained the ascending response values, a second the descending values and a third dataset was compiled from the shuffled response values. Summarizing the data set up, nine sets with molecules, each set compiled three descriptors, gave 27 descriptor matrices ***X***. Three different response values, molecular weight, cLogP, and the number of sp3-carbon atoms were sorted according to ascending, descending or shuffled data. Combined with the 27 ***X*** matrices, a total of 243 datasets were obtained. The molecules together with the descriptors and the calculated response values are available from (Korff, 2021). Each of these datasets underwent the successive regression procedure, as described in the next two paragraphs.

### Machine Learning Techniques

Six modeling techniques were applied to construct regression models for the datasets: *k* next neighbor regression (*k*NN), partial least square regression (PLS), partial least square regression with power transformation (PLSP), random forest regression (RFR), Gaussian process regression (GPR), and support vector (SVM) regression. All parameters for these machine learning models were optimized by an exhaustive search. The median model was used as a baseline model. Any successful machine learning model should be significantly better than the baseline model. Also easy to calculate was the *k* next neighbor model for regression. In this model, the *k* next neighbors in the training set were screened for the query descriptor vector. The predicted $\hat{y}$ value was the average of the corresponding *y* values weighted by similarity. Partial least square regression (PLSR) is a multivariate linear regression technique (De Jong, 1993), which only requires the number of factors as the input parameter. PLSR with power transformation includes a Box Cox transformation and is often used to model biological data, which are notoriously not normally distributed (Sakia, 1992). For random forests and Gaussian process regression, we used the implementation from Haifeng Li. (Li, 2021). Random forest regression was only included because it is frequently used for models in drug discovery. Random forests base on decision trees and are not capable of extrapolation. The Java program library libsvm was used for the support vector machine regression (Chang and Lin, 2011). Details for meta-parameter search: *k*NN: *k* from 1 to 9, step 1. PLSR: factors from 1 to 31, step 1. PLSR power transformation: factors like PLSR; *λ* from 0.05 to 2, step 0.05. Gaussian process regression: *λ* 0.001, 0025 … 1, … 10,10,000. Random forest: trees 50, 100, 250, 500, 1,000; Maximum number leaf nodes from 2 to 54 step 2. Mtry: 0.15, 0.333, 0.45. Maximum node size from 2 to 54, step 2. Support vector regression: (Smola and Schölkopf, 2004) Epsilon regression, RBF kernel, power of 2 rule for: C from 2 to 5 to 215; *ϵ* from 2 to 10 to 26; *γ* 1/(number of fields in the descriptor). Details for the objective function are given in the next section.

### Successive Regression

A two-step process was implemented to ensure an unbiased estimation for the extrapolation power of a model. The first step was the selection of one meta-parameter set for every machine learning technique. The algorithm started with the first 20% of the molecule descriptors ***X***_0,0.2_, ***y***_0,0.2_ together with the measured response values to determine the meta parameters of the machine learning models *via* an exhaustive search. An eleven-fold Monte Carlo cross validation was employed to split all data into the training and validation datasets (Xu and Liang, 2001). A left out fraction of 25% was chosen as the size of the validation dataset. With this set up, the average error for all meta-parameter sets was calculated. For each machine learning technique ***t***, the meta-parameter set ***M***_min,*t*_ was chosen that showed the minimum average error. This meta-parameter set was used to construct a model from all data in ***X***_0,0.2_, ***y***_0,0.2_. In the second step, an independent test set was compiled from the next 10% of data, ***X***_0.3_, ***y***_0.3_. The average prediction error of $\hat{y_{0.3}}$ gave an unbiased estimator for the model, because the machine learning algorithm ***M***_min,*t*,0.2_ had not seen these data before prediction. Subsequently, step one was repeated, this time with the dataset ***X***_0.3_, ***y***_0.3_. So, the former test data were added to ***X***_0,0.2_, ***y***_0,0.2_. The meta parameter for the machine learning algorithms ***M***_min,*t*,0.3_ were now determined with ***X***_0,0.3_, ***y***_0,0.3_. So, the prediction was done for ***y***_0.4_. This process was repeated eight times, up to a model size with ***X***_0,0.9_, ***y***_0,0.9_ and a prediction for ***y***_1.0_. Using this method, we assessed the extrapolation power of the machine learning method together with the applied molecular descriptor for the sorted response data. The 10% test set, with higher or lower response values than the training set, was an unbiased estimator of the model’s quality for extrapolation. As a quality measure for prediction, we used the relative error.

### Technical Details

The source code was implemented in Java 1.8. The calculations were done on a SuperMicro computer with 176 processor cores. Meta-parameter calculation and test data prediction took approximately 72 h for all datasets. Data visualization was done with DataWarrior (Sander et al., 2015), an open source tool for data visualization and evaluation (Sander, 2021).

## Results

**FIGURE 3:**
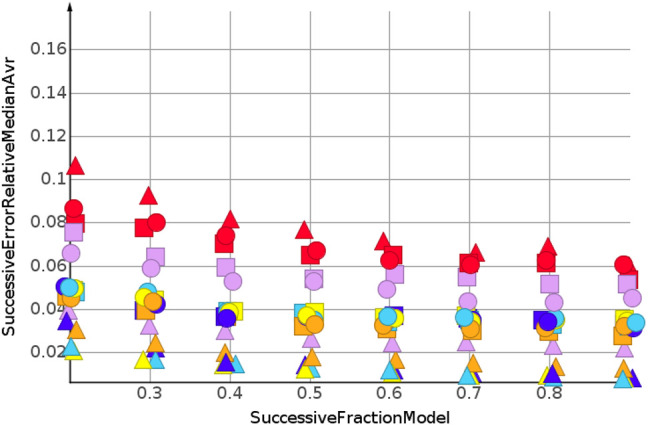
Prediction of molecular weight, random shuffling.

The successive regression procedure was applied to all 243 datasets. In the following, the results for nine datasets with the molecular structures ***S***_***apix,1–3***_, ***S***_***rosu,1–3***_ and ***S***_***sofo,1–3***_ are summarized by their median relative error. No extrapolation was needed for the shuffled datasets. Figure 3 and Table 2 show the machine learning results for the prediction for the shuffled data for three descriptors and three properties. The three descriptors, fragment fingerprint, path fingerprint and skeleton spheres, are indicated by shape. Circles, squares and triangles indicate fragment fingerprint, path fingerprint and skeleton spheres descriptors, respectively. A color code was used for the machine learning algorithms. Green indicated our base line model, which was the prediction by median, *k*NN regression in red, Gaussian process regression in blue, partial least square regression in yellow, partial least square regression with power transformation in light blue, random forest regression in magenta, and support vector regression in orange. All results are available as Data Warrior files (Korff, 2021).

**TABLE 2:** Prediction of molecular weight, random shuffling, skeleton spheres descriptor. The first column indicates the machine learning algorithm. The first row is the fraction of data used for model construction. The other values are the relative errors of the test data.

	Fraction of train data						
	0.30	0.40	0.50	0.60	0.70	0.80	0.90
GPR	0.02	0.01	0.01	0.01	0.01	0.01	0.01
Med	0.29	0.30	0.30	0.29	0.29	0.29	0.28
PLS	0.02	0.01	0.01	0.01	0.01	0.01	0.01
PLSP	0.02	0.01	0.01	0.01	0.01	0.01	0.01
RFR	0.03	0.03	0.02	0.02	0.02	0.02	0.02
SVM	0.02	0.02	0.02	0.02	0.01	0.01	0.01
*k*NN	0.09	0.08	0.08	0.07	0.06	0.07	0.06

For almost all models, the relative error for predicted molecular weight was less than 10%. For the majority of predictions, relative error was less than or equal 5%. No preference for any of the descriptors was observed, as indicated in Figure 3. A higher separation was shown by the machine learning techniques. The error for the median model is not shown in Figure 3. A relative error of approximately 30% was observed for all fractions of the model. Three machine learning models performed equally well. Gaussian process regression, partial least square regression and partial least square regression with power transformation showed a relative error below 3%. These results were obtained together with the skeleton spheres descriptor.

**FIGURE 4:**
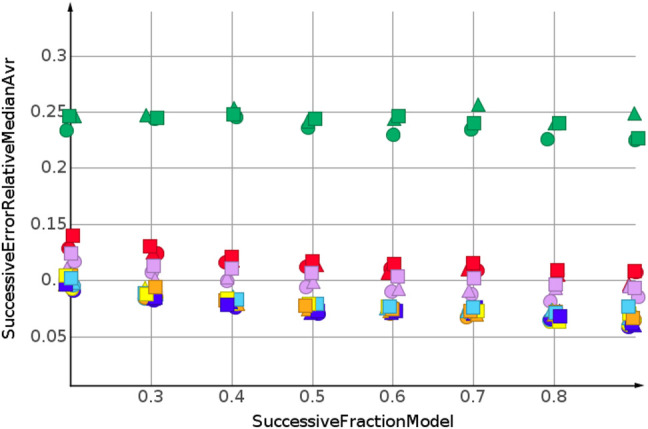
sp3-atoms, random shuffling.

Figure 4 shows the results for the sp3-atoms with the shuffled data. The results were similar to the predicted molecular weight in 3. Relative error was higher than for the molecular weight prediction, but all models were better than the median model. In contrast to the molecular weight prediction, all three descriptors performed equally well for the models with the lowest error.

**FIGURE 5:**
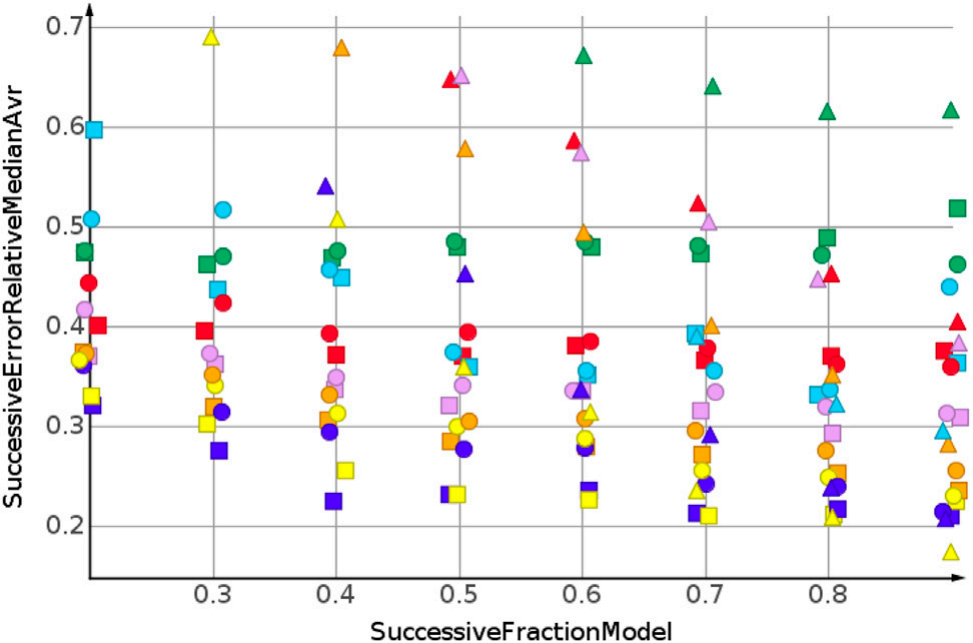
cLogP, random shuffling.

The predictions for cLogP, draw a different picture than the predictions for molecular weight and number of sp3-atoms, Figure 5. Only one model showed a relative error below 20%. Many models were worse than the median model, indicated in green. The best performing machine learning models were partial least square regression and Gaussian process regression.

**FIGURE 6:**
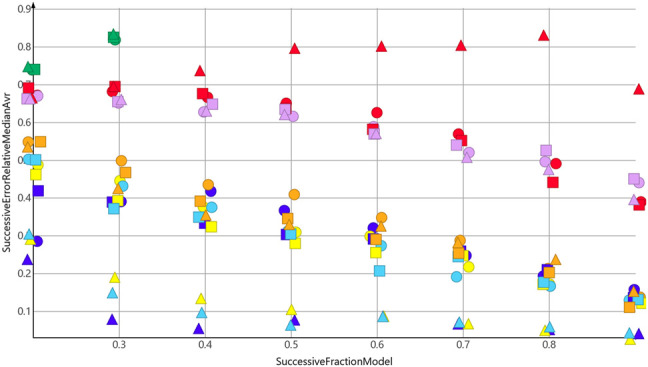
Molecular weight, high to low sorted response values.

The prediction for shuffled data did not require extrapolation. The data range of the response values is covered by the training data. To simulate the requirements of drug discovery, the datasets were sorted by their response values. In the following, we discuss the results for sorting from high to low response values. This experimental set up forced the machine learning algorithms into extrapolation. The range of predicted response values was always outside the range of the training data. Figure 6 shows the results for the prediction of molecular weight. The data were sorted from high to low.

As for the molecular weight prediction for shuffled data, the skeleton spheres descriptor together with partial least square regression, partial least square regression with power transformation and Gaussian process regression delivered the most predictive models. The range of the relative errors was very large, below 10% for the best models up to 50% for the *k*NN models with the skeleton spheres descriptor, depicted in red triangles. But, relative errror was higher for all predictions than for the shuffled data. For the shuffled data, only one prediction was above a relative error of 0.1, with the *k*NN model at a fraction of 0.2. For the high to low sorted molecular weight data the majority of predictions showed a relative error above 0.1.

**FIGURE 7:**
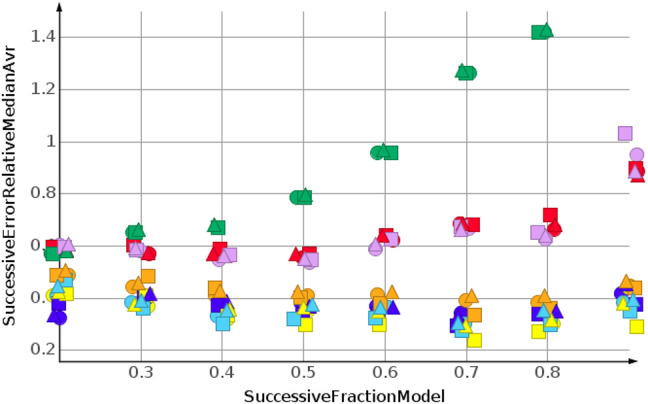
Number of sp3-atoms, high to low sorted response values.

Two trends were observed for the prediction of number of sp3-atoms, Figure 7. The relative error of the median prediction increased with an increasing fraction of data used to construct the models. This also happened with the relative error for the *k*NN models, in red, and the random forest, in magenta. The relative error for Gaussian process regression, partial least squares, partial least squares with power transformation and support vector regression remained almost constant. As for molecular weight, the predictions for high to low sorted data had a much higher relative error than predictions for sorted data.

**FIGURE 8:**
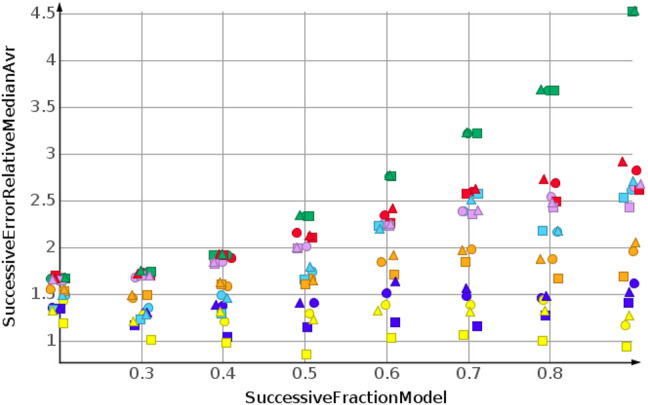
cLogP, high to low sorted response values.

Figure 8 shows the results for cLogP. Data were sorted from high to low. Curve progression was similar to the curve progression of the relative error for the sp3-atom number prediction. However, the values for the relative error are much higher. Only four predictions had relative errors less than 100%.

**FIGURE 9:**
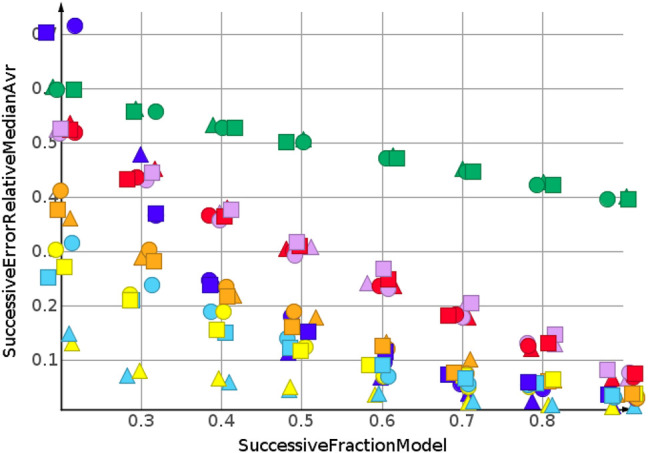
Molecular weight, low to high sorted response values.

**FIGURE 10:**
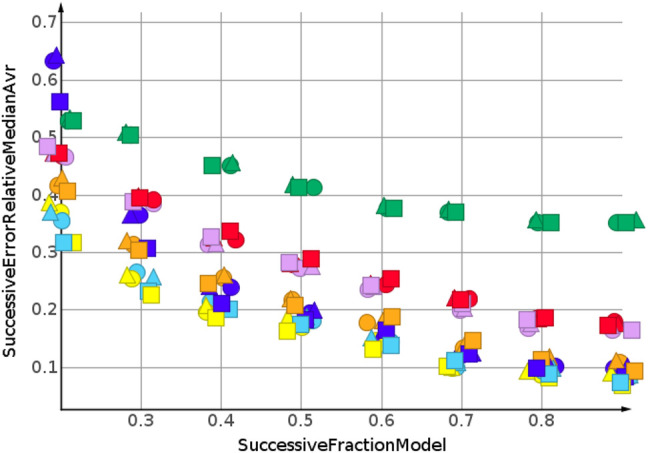
Number of sp3-atoms, low to high sorted response values.

**FIGURE 11:**
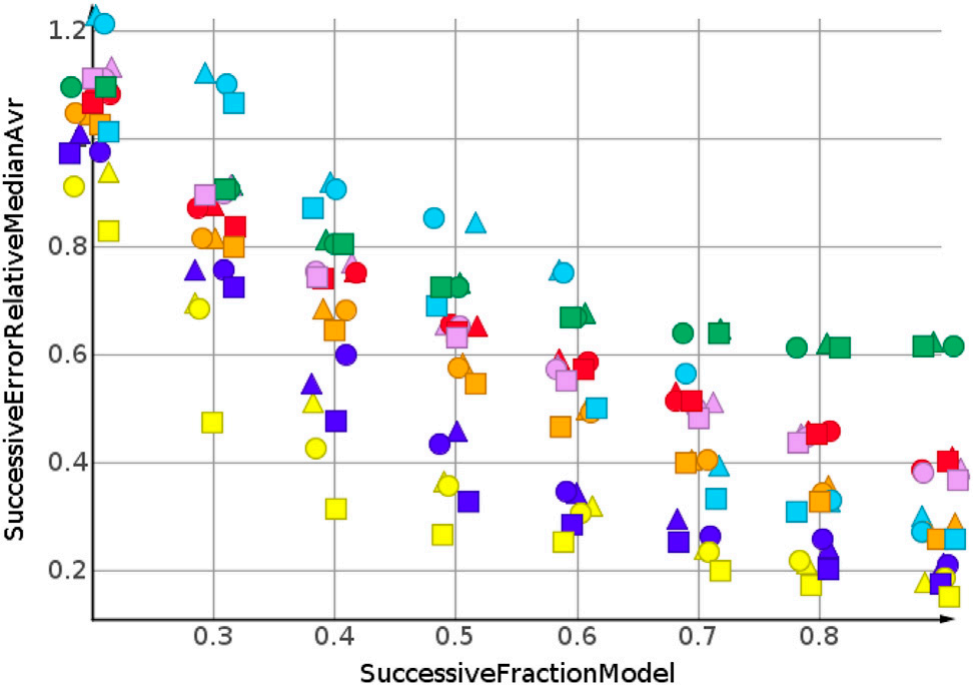
cLogP, low to high sorted response values.

When molecular weight values were sorted from low to high, the values to be predicted were higher than the values used for model construction. For the molecular weight prediction, the results are depicted in Figure 9. The skeleton spheres descriptor resulted in models with the lowest relative error. In the figures, the skeleton spheres descriptor is indicated by triangles. For the prediction of the number of sp3-atoms in Figure 10 the models constructed from the path fingerprint were better than the models constructed from the skeleton spheres descriptor. As for the high to low sorted values in Figure 7, the path fingerprint was the best performing descriptor. Also, for cLogP value prediction, given in Figure 11, the path fingerprint was the best performing descriptor.

For each of the experimental set ups, including 243 individual datasets, all machine learning algorithms outperformed median predictions, which were used as baseline controls. *k*NN regression and random forest regression were very similar in their prediction quality. These two algorithms were outperformed by support vector regression. The best performing machine learning algorithms were Gaussian process regression, partial least square regression and partial least square regression with power transformation. Together with the path fingerprint and the skeleton spheres descriptor, the best results were obtained. The relative errors for the successive predictions were lower for the low to high sorted values than for the high to low sorted values. This was caused by numeric effects, the absolute prediction error for big values results in a lower relative error than the same absolute prediction error for small values.

## Results Summary and Conclusion

All results are summarized in Tables 3–5. The figure of merit was the rank of the median error. For every successive fraction of test data, a median error was calculated from the nine molecule datasets ***S***_***apix,1–3***_, ***S***_***rosu,1–3***_ and ***S***_***sofo,1–3***_. By using the ranks of the errors, a bias was prevented, which would have been otherwise introduced by the error dependency on the fraction of training data. Because, a higher fraction of training data generally results in better models. This would have resulted in a bias if the median would have been used. By using the ranks the results for different fractions of training could be combined. In Tables 3–5, the frequency of the top three ranks is given. This means, the rank count increased by one, if the corresponding error belonged to the three lowest errors for the given conditions. Results for the machine learning algorithms are provided in Table 3. For shuffled response data, Gaussian process regression delivered the highest number of top models 9) for prediction. For extrapolation, for high to low sorted and for low to high sorted data, the partial least square regression outperformed the other machine learning algorithms. That the linear method outperformed the non-linear method is in accordance with the results from (Cortés-Ciriano et al., 2018), where the linear method, ridge regression, also outperformed the non-linear method, random forest.

**TABLE 3:** Summary of the best results for the machine learning techniques. Rank count for the top three ranks. The ranks were calculated from all descriptors, predicted properties, and fractions of training data. The columns show the three different orientations of the response data: shuffled, sorted from high to low and from low to high.

ML method	Shuffled	low2high	high2low
Gaussian process regression	9	6	1
KNN regression	0	0	0
Median	0	0	0
PLS	8	13	18
PLS Power	3	6	6
Random Forest regression	0	0	0
SVM regression	7	2	2

Results for the descriptors are provided in Table 4. In total, the skeleton spheres descriptor outperformed the other two descriptors. However, the path fingerprint slightly outperformed the skeleton spheres descriptor for extrapolation for the high to low sorted response values. Table 5 presents the rank counts for the most accurately predicted response values. As expected, the best models were obtained for molecular weight, followed by the number of sp3-atoms.

**TABLE 4:** Summary of the best results for the three descriptors. Rank count for the top three ranks. The ranks were calculated from all methods, predicted properties, and fractions of training data.

Descriptor	Shuffled	low2high	high2low
FragFp	17	16	13
PathFp	16	22	26
SkelSpheres	30	25	24

**TABLE 5:** Summary of the best results for the three response datasets. Rank count for the top three ranks. The ranks were calculated from all descriptors, methods, and fractions of training data.

Response value	Shuffled	low2high	high2low
MW	54	47	49
cLogP	0	0	0
sp3-Atoms	9	16	14

The purpose of this study was to examine the difference between prediction in the range of the training response values and extrapolation outside the training response values. It must be considered that the molecules in each dataset were derived from a single molecule. Consequently, there was a high similarity between molecules in a dataset. All molecules in this examination were in the domain of applicability. They were similar to the training molecules in descriptor space. Nevertheless, the differences between the relative errors for the shuffled data and sorted data were striking. Even for molecular weight, with a very low error for shuffled data, the extrapolation for high to low sorted data became much more difficult. This was unexpected, because molecular weight depends solely on the molecular formula and does not need any molecular graph dependent feature. In addition, the relation between the molecular formula and molecular weight is strictly linear. cLogP values were hardest to predict. Prediction was achieved with a moderate error for shuffled data using linear regression techniques. However, after sorting the response values from high to low and successively extrapolating the lower values, no meaningful prediction for cLogP was possible. None of the machine learning algorithms were able to extrapolate cLogP values for high to low sorted data. This result was unexpected because the cLogP model is an incremental model that relies on substructure contributions to the overall cLogP. Therefore the contributions are linear and theoretically can be modelled by linear regression with chemical descriptors. We had expected, that the linear regression algorithms would be able to create “semi-mechanistic” models with more predictive power. There is a high demand in drug discovery for extrapolation of molecular features. The results of this study show large differences in prediction quality between interpolation and extrapolation. This demonstrates that any model used for extrapolation should be validated with extrapolation. For this validation, we suggest the successive prediction as described in this contribution. We suggest to add the prediction of calculated values as reference standard to all publications in cheminformatics when regression methods are applied. Partial least square regression was by far the most successful extrapolation method. The successful extrapolation of molecular features show that partial least square regression is capable of providing meaningful models for extrapolation.

## Data Availability

The original contributions presented in the study are included in the article/Supplementary Materials, further inquiries can be directed to the corresponding author.
